# Plumbagin can potently enhance the activity of xanthine oxidase: *in vitro*, *in vivo* and *in silico* studies

**DOI:** 10.1186/s40360-021-00511-z

**Published:** 2021-07-17

**Authors:** Liang Yue, Nan Jiang, Anguo Wu, Wenqiao Qiu, Xin Shen, Dalian Qin, Hong Li, Jing Lin, Sicheng Liang, Jianming Wu

**Affiliations:** 1 Department of Pharmacology, School of Pharmacy, Southwest Medical University, Luzhou, 646000 Sichuan China; 2 Institute of Cardiovascular Research, the Key Laboratory of Medical Electrophysiology, Ministry of Education of China, Medical Key Laboratory for Drug Discovery and Druggability Evaluation of Sichuan Province, Luzhou Key Laboratory of Activity Screening and Druggability Evaluation for Chinese Materia Medica, Luzhou, 646000 Sichuan China; 3 Department of Gastroenterology, The Affiliated Hospital of Southwest Medical University, Luzhou, 646000 Sichuan China

**Keywords:** Xanthine oxidase, Plumbagin, Uric acid, Enzymatic reaction kinetics, Toxic metabolism

## Abstract

**Background:**

Abnormally elevated xanthine oxidase (XO) activity has been verified to cause various pathological processes, such as gout, oxidative stress injury and metabolic syndrome. Thus, XO activators may exhibit above potential toxicological properties. Plumbagin (PLB) is an important active compound in traditional Chinese medicine (TCM), while its obvious toxic effects have been reported, including diarrhea, skin rashes and hepatic toxicity. However, the potential toxicity associated with enhancement of XO activity has not been fully illuminated so far.

**Methods:**

The present study investigated the effect of PLB on XO activity by culturing mouse liver S9 (MLS9), human liver S9 (HLS9), XO monoenzyme system with PLB and xanthine. Then, the molecular docking and biolayer interferometry analysis were adopted to study the binding properties between PLB and XO. Finally, the in vivo acceleration effect also investigated by injected intraperitoneally PLB to KM mice for 3 days.

**Results:**

PLB could obviously accelerate xanthine oxidation in the above three incubation systems. Both the V_max_ values and intrinsic clearance values (CL_int_, V_max_/K_m_) of XO in the three incubation systems increased along with elevated PLB concentration. In addition, the molecular docking study and label-free biolayer interferometry assay displayed that PLB was well bound to XO. In addition, the in vivo results showed that PLB (2 and 10 mg/kg) significantly increased serum uric acid levels and enhanced serum XO activity in mice.

**Conclusion:**

In summary, this study outlines a potential source of toxicity for PLB due to the powerful enhancement of XO activity, which may provide the crucial reminding for the PLB-containing preparation development and clinical application.

## Background

Xanthine oxidase (XO) is a complex metalloflavoprotein enzyme which mainly distributing in the liver and intestine of mammals [1]. It is one of the most important enzymes in nucleic acid metabolism [2] that directly regulates the production of uric acid in vivo [3]. Besides, XO can also metabolize a wide variety of compounds with heterocyclic systems, such as purines, pyridines, pyrimidines and their structural analogues, plays significant roles in many physiological processes [4].

Due to the critical role of XO in purine metabolism, XO has been developed as an important drug target. As the final product of the purine compound metabolic pathway, uric acid exists mainly as monosodium urate (MSU) crystallization with low solubility in biological environments, its content rises with elevated activity of XO [5]. When the MSU concentration exceeds the maximum solubility in the body, it easily precipitates and deposits in the joint cavity, eventually leading to gout, a clinical issue associated with hyperuricaemia [6].

In fact, XO-catalysed xanthine oxidation can not only affect the formation of uric acid but also release a large number of oxygen radicals and hydrogen peroxide molecules in the process of oxidation [7]. Therefore, abnormal increases in XO activity leads to oxidative stress injury [8], metabolic syndrome and inflammation [9]. From this perspective, XO can even be regarded as a toxic target. Therefore, the potential side effects of drugs similar to XO activators are not negligible. Unfortunately, this phenomenon did not attract the attention of researchers.

**Fig. 1:**
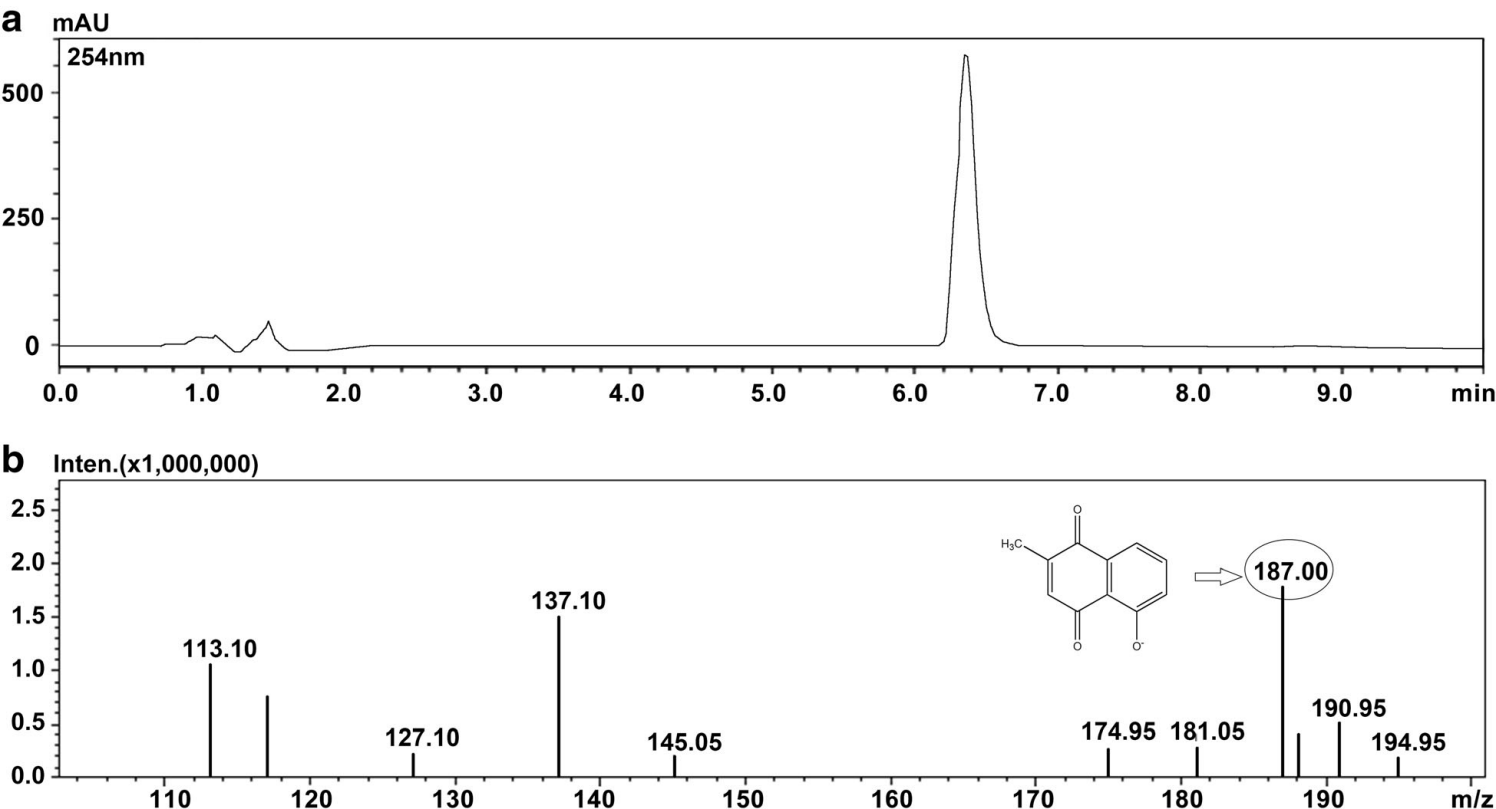
**A** The HPLC chromatogram of PLB. **B** The precursor ion spectra in negative ionization model of PLB

Plumbagin (PLB, Fig. 1) is a traditional Chinese medicine (TCM) component which derived from the root of *Plumbago zeylanica* L, it has been verified to show a wide range of pharmacological effects, such as anticancer, antibiosis and antifungal [10–12]. On the other hand, the toxicity of TCM has always been payed much attention, which is the primary reason for limiting the use of TCM. Without exception, a large number of studies about the toxicity of PLB have been reported, including cardiovascular diseases [13] and hepatic toxicity [14]. However, we don’t know if these toxicities are associated with elevated XO activity, although increasing evidences show that PLB is actively involved in redox cycling [15], few details are known.

In this study, we found that PLB showed a strong activation effect on XO. To investigate the interaction between PLB and XO, we subsequently verified the activation of PLB on XO in three enzyme reaction systems, including MLS9, HLS9 and XO monoenzyme in vitro by using xanthine as a marker substrate. Then, molecular docking and biolayer interferometry analysis were adopted to study the binding properties between PLB and XO. Finally, the effect of PLB on uric acid level and serum XO activity in mice were also investigated. It is hoped that this study will help us to better understand the toxicological properties of PLB.

## Methods

### Reagents

Plumbagin (purity > 99%) was purchased from Tauto Biotech (Shanghai, China). Xanthine and LC-MS grade acetonitrile and formic acid were purchased from Damas-beta (Shanghai, China). Uric acid was purchased from Tokyo Chemical Industry Co., Ltd. (Tokyo, Japan). RLS9 and HLS9 were purchased from the research institute for liver diseases (RILD, Shang Hai, China), and XO monoenzyme (from bovine milk) were purchased from Sigma (St. Louis, Missouri, USA). Phosphate buffered saline (PBS) was obtained from Solarbio (Beijing, China). Dimethyl sulfoxide (DMSO, purity > 99.9%) was obtained from Yatai United Chemical Co., Ltd. (Wuxi, China).

### Animal studies

Kunming (KM) mice (both sexes), weighing 25–30 g, were procured from the experimental animal center of southwest medical university (Luzhou, China). Mice were housed in well-ventilated cages at room temperature (25 ± 2 °C) with 40–60% relative humidity on a regular 12 h light-dark cycle. The animals were acclimatized for a minimum period of 3 days prior to the experiment.

### Incubation conditions

General incubation conditions were maintained in a reaction mixture of 100 μL of 50 mM PBS buffer (pH = 7.4) containing xanthine as the substrate. Prior to reaction initiation by adding MLS9, HLS9 or XO, all the reaction mixtures were pre-incubated at 37 °C for 5 min. The optimal incubation time for each system depended on the production of uric acid at different time points. Then, the reaction was terminated by denaturing the protein with high-temperature heating at 95 °C in a dry thermostat (Haimen Kylin-Bell Lab Instruments Co, Ltd., Haimen, China). Finally, the mixtures were centrifuged at 20000 g for 15 min, and the supernatants were analysed by HPLC to detect the formation of uric acid.

### Kinetic assays for stimulation of uric acid formation by PLB in MLS9, HLS9 and XO monoenzyme system

In this study, various concentrations of xanthine (5–480 μM, covering a K_m_ value of uric acid production in the three incubation systems) and PLB (0–80 μM, covering the level displaying half maximal stimulation in the three incubation systems) were co-incubated with MLS9 (0.2 mg/mL), HLS9 (0.2 mg/mL) or XO (0.005 U). All incubations were performed at 37 °C for 10 min (MLS9), 30 min (HLS9), and 5 min (XO) in duplicate measurements. Apparent kinetic parameters were calculated as the mean ± S.E.

### Effect of PLB on KM mice

In this study, all mice were randomly divided into four groups (*n* = 6). Ten milligram per kilogram PLB was dissolved in DMSO (2%), ethanol (5%), tween-80 (5%) and physiological saline (88%), then dilute to 2 mg / kg with the same solvent system. The normal control mice were treated with a solvent vehicle, and febuxostat-treated mice were set as the negative control group at the same time. All mice were injected intraperitoneally for 3 days. One hour after the last dose, the eyeballs of all mice were removed for blood collection, and the blood samples were kept overnight at 4 °C to completely solidify. Then, all samples were centrifuged at 3000 g for 10 min to obtain serum. A total of 10 μL of each serum sample was incubated with the substrate at 37 °C for 60 min, while the same amount of serum was then dissolved in PBS, and the uric acid content of the samples was detected to eliminate the interference of endogenous uric acid. The production rate of uric acid was finally calculated to evaluate the activity of XO in each serum sample. On the other hand, 100 μL of each serum sample was transferred into a 1.5 mL eppendorf tube, and 400 μL of hydrochloric acid (2 M) was added. The mixture was vortexed for 2 min. After centrifugation at 14,000 rpm for 10 min, the supernatant was transferred into another tube, and then all the samples were freeze-dried (SCIENTZ-12 N). Finally, the residue was dissolved in 100 μL of initial mobile phase by vortexing for 5 min and sonicating for 10 min, and further centrifugation at 14000 rpm for 10 min was applied after the residue was redissolved. The supernatant was injected into the UHPLC system for analysis.

### Data analysis

The mean and SE values of all incubation systems were calculated. The apparent kinetic parameters of xanthine oxidation (K_m_ and V_max_) in the absence or presence of PLB were calculated by combining the kinetic equation of the apparent formation rate of uric acid with nonlinear regression analysis software (GraphPad Prism Software Inc., Version 5, San Diego, CA). *p* < 0.05 was considered statistically significant. Kinetic parameters of xanthine oxidation stimulation by PLB in all incubation systems (MLS9, HLS9 and XO) were described by fitting the data with the Michaelis–Menten model.

### HPLC analysis

Xanthine oxidation samples were analysed by a Shimadzu (Kyoto, Japan) Prominence UFLC (Ultra-fast liquid chromatography) system consisting of two LC-20 AD pumps, a SIL-20ACHT autosampler, a CBM-20A communications bus module, a SPD-M20A diode array detector, a CTO-20 AC column heater-cooler, and a reversed-phase XCharge-C_18_ column (4.6 × 150 mm, 5 μL, Acchrom Trchnologies Co, Ltd., Beijing, China), which was used for the analysis and maintained at 30 °C. The mobile phase consisted of acetonitrile (Solvent A) and 0.1% formic acid in water (Solvent B), and the flow rate was 1 mL/min. A 6.5 min isocratic elution programme comprised of acetonitrile:0.1% formic acid in water (5:95, v/v) was used at a flow rate of 1 mL/min, and the injection volume was 10 μL.

### Molecular docking studies

In this study, molecular docking was performed using AutoDockTools-1.5.6, and the crystal structure of XO (PDB: 2E1Q) was obtained from the Protein Data Bank (http://www.rcsb.org/pdb/). The 3D structure of PLB was retrieved from the PubChem database. The binding pocket was searched using the Biovia drug discovery studio visualizer 2020. The structure of XO was then treated by removing water molecules, adding hydrogens and removing ligands. Kollman united atom charges, solvation parameters and polar hydrogen atoms were added to XO for the preparation of the docking simulation. The docking operation followed the lamarck genetic algorithm, and all other docking and scoring parameters were set to their default values. The best conformation was selected according to the binding free energy and inhibition constant and then put into PyMOL software for visualization.

### Biolayer interferometry analysis

First, 200 μL of XO (200 μM) was prepared for use. EZ-Link NHS-LC-LC-Biotin (Thermo Scientific, United States) was prepared with DMSO as the working solution at a 10 mM concentration. XO and biotin were incubated at 4 °C for 4 h at a molar ratio of 1:3. Then, the biotinized XO was filtered through a zeba desalination column. The filtered solution was collected and incubated overnight in an octagonal tube at 4 °C to fix the biotinylated XO protein to a streptavidin (SA) (ForteiBIO, Menlo Park, CA, United States) sensor. Then, the SA sensor with biotinized XO was balanced in a PBS (containing 5% DMSO) solution. At the same time, the prewetted SA biosensor was used to record the baseline. Finally, 200 μL of PLB (0, 25, 50, 100 and 200 μM) was added to a 96-well plate, and the concentration of DMSO was adjusted to be consistent with that of the control group. All the experiments were composed of three main steps: baseline (60 s), combination (50 s) and dissociation (50 s). The combination and dissociation curves and kinetic constants were obtained by using ForteiBIO data analysis software.

## Results

### Establishment of incubation system

**Fig. 2:**
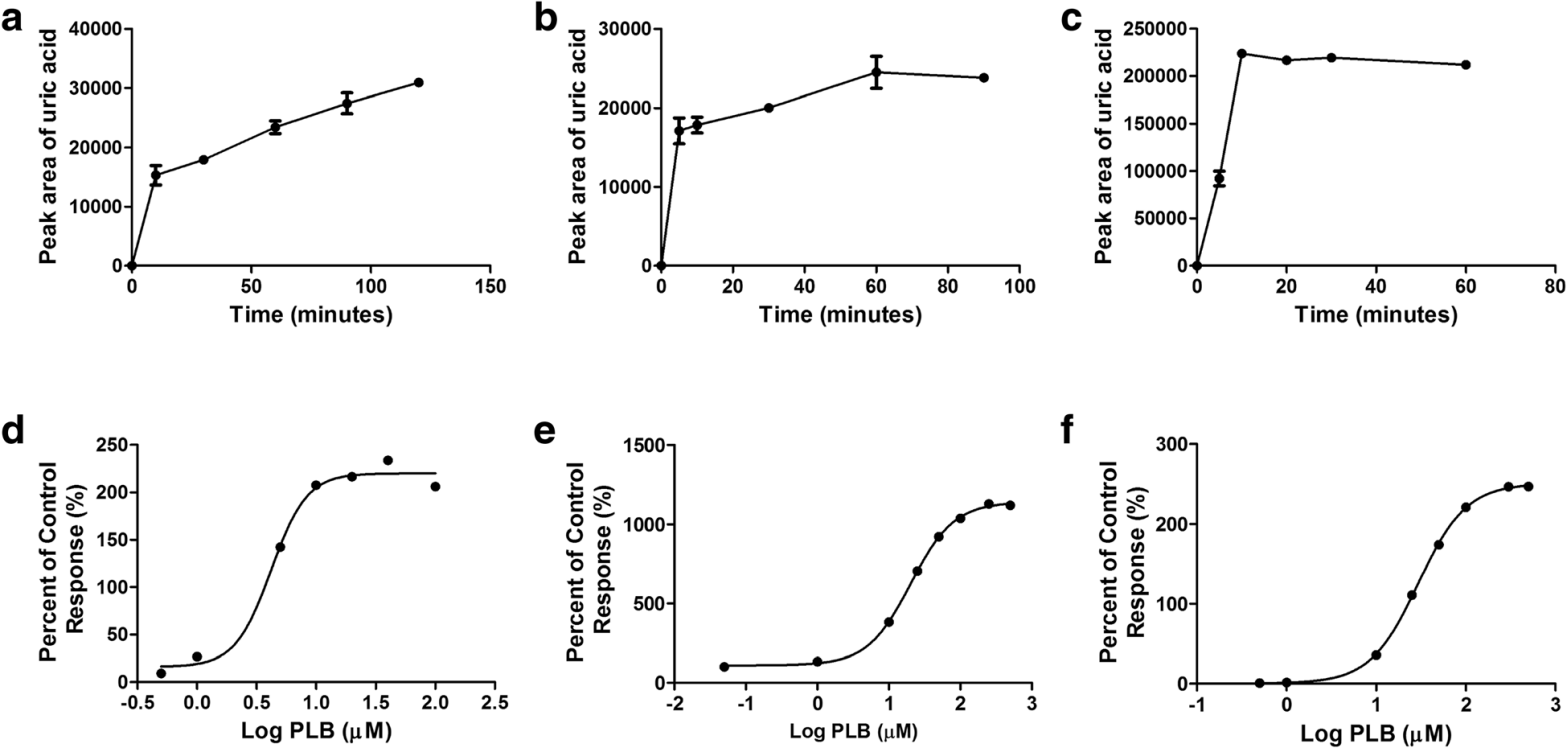
Confirmation of optimal incubation conditions. **A, B, C** Variations in the production of uric acid over time in MLS9, HLS9 and XO, respectively. Each point and error bar represents the average and S.D. of duplicate measurements. **D, E, F** The EC_50_ values of PLB in MLS9, HLS9 and XO for the activation of XO. Each point represents the average of duplicate measurements

To establish an efficient reaction incubation system for xanthine oxidation, we monitored the incubation time and finalized the optimal incubation time for the MLS9, HLS9, and XO systems (60, 30, and 5 min, respectively) (Fig. 2A-C). In addition, the EC_50_ values of PLB for the activation of xanthine oxidase in the above three systems were also measured as 4.19, 29.51, and 20.22 μM, respectively (Fig. 2D-F).

### Kinetic analyses for the stimulation of uric acid production in MLS9

**Fig. 3:**
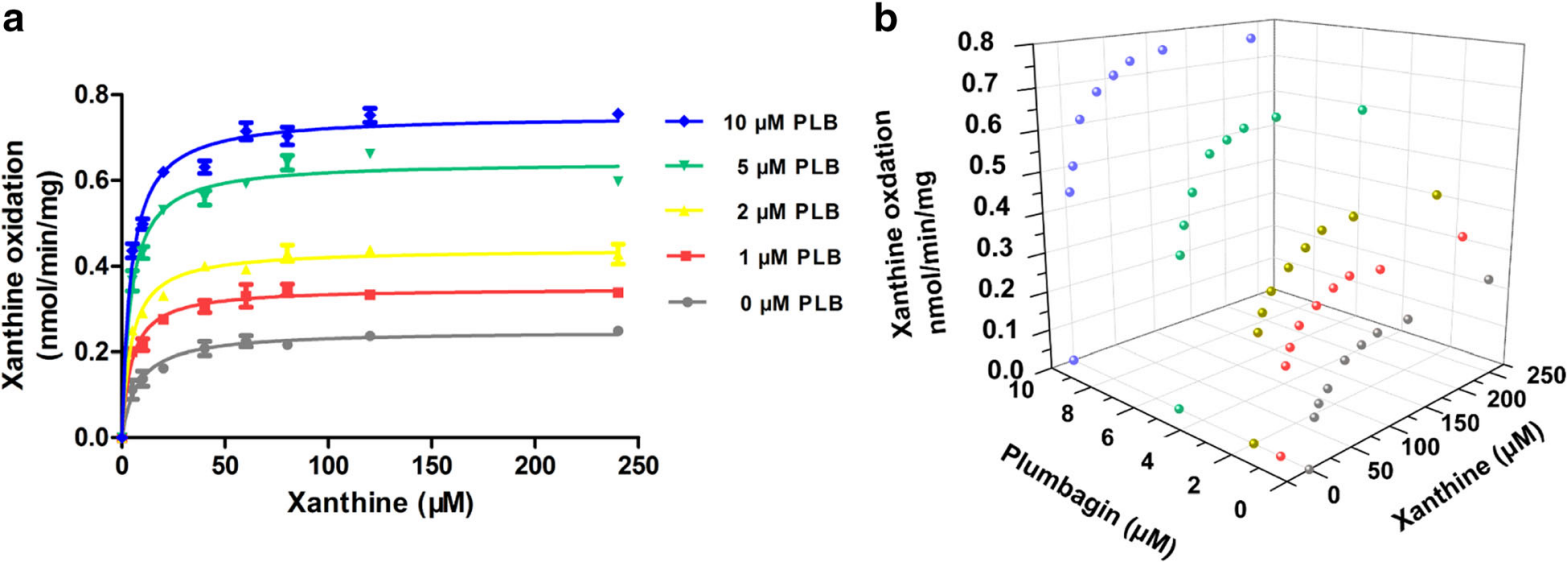
Kinetic analyses for xanthine oxidation stimulation in MLS9 in the presence of various concentrations of PLB. **A** Kinetics of xanthine oxidation in MLS9 in the presence of various concentrations of PLB. Each point and error bar represents the average and S.D. of duplicate measurements, respectively, and the curves were drawn by fitting the Michaelis–Menten equation to the data. **B** 3D scatter graph of xanthine oxidation kinetics caused by PLB in MLS9. Each point represents the average of duplicate measurements

We first carried out a kinetic analysis of PLB stimulation of uric acid production in the MLS9 system. Various concentrations of PLB could significantly accelerate the formation of uric acid at all xanthine concentrations (Fig. 3A). Xanthine oxidation was in accordance with the Michaelis-Menten model at various PLB concentrations (Fig. 3A and Table 1). In the presence of all PLB concentrations employed in this study, the K_m_ values decreased, while the V_max_ values and intrinsic clearance values (CL_int_, V_max_/K_m_) of xanthine oxidation increased in a PLB concentration-dependent manner, ranging from 0.248 to 0.753 nmol/min/mg and 32 to 175 μL/min/mg, respectively (Table 1). The data were visualized in the form of a 3D scatter graph at the same time (Fig. 3B).

**Table 1:** Kinetic parameters for xanthine oxidation in MLS9 system. Constants are expressed as the mean ± SE

PLB μM	K_**m**_ μM	V_**max**_ nmol/min/mg	Goodness of fit R^**2**^	CL_**int**_ μL/min/mg
0	7.8 ± 1.0	0.248 ± 0.006	0.972	32
1	4.7 ± 0.5	0.349 ± 0.006	0.980	74
2	4.6 ± 0.5	0.440 ± 0.007	0.984	96
5	4.3 ± 0.5	0.644 ± 0.011	0.983	150
10	4.3 ± 0.4	0.753 ± 0.011	0.987	175

### Kinetic analyses for the stimulation of uric acid production in HLS9

**Fig. 4:**
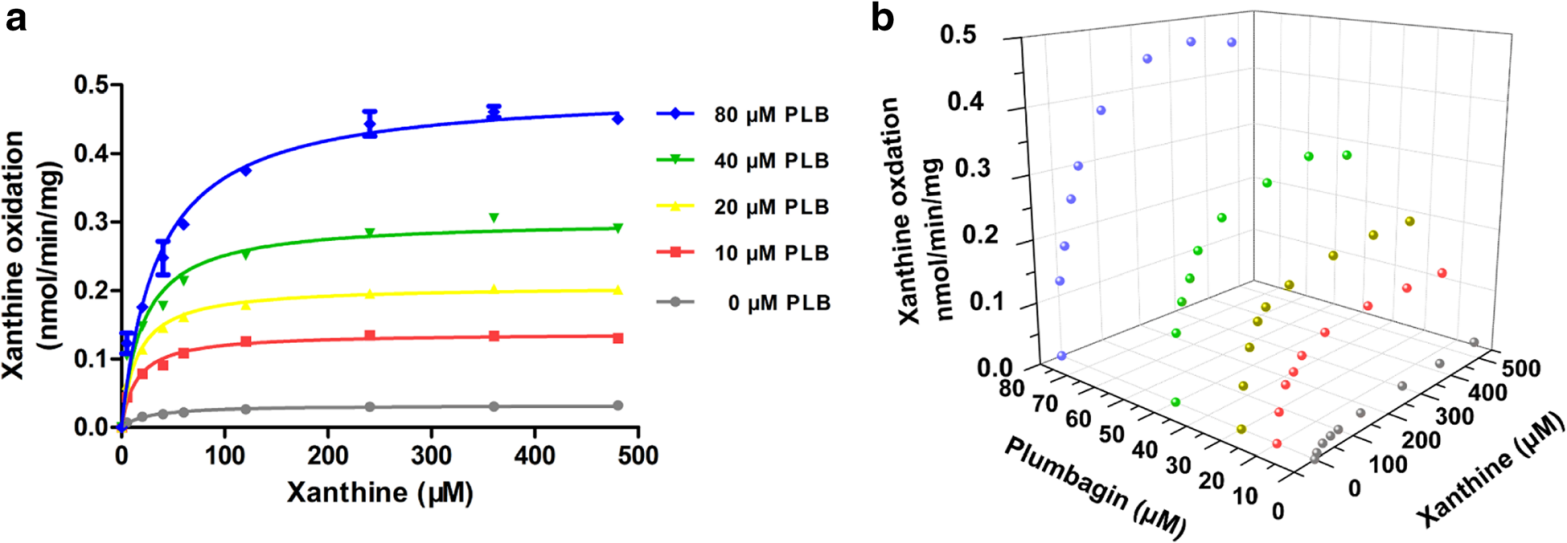
Kinetic analyses for xanthine oxidation stimulation in HLS9 in the presence of various concentrations of PLB. **A** Kinetics of xanthine oxidation in HLS9 in the presence of various concentrations of PLB. Each point and error bar represents the average and S.D. of duplicate measurements, respectively, and the curves were drawn by fitting the Michaelis–Menten equation to the data. **B** 3D scatter graph for kinetics of activation xanthine oxidation by PLB in HLS9. Each point represents the average of duplicate measurements

To explore whether PLB also stimulated xanthine oxidation in HLS9, kinetic analyses were conducted for the reaction in the presence of various PLB concentrations as well (Fig. 4A and Table 2). In the presence of PLB concentrations of 0, 10, 20, 40, and 80 μM, the K_m_ values were 25.2, 14.7, 15.3, 20.7, and 35.2 μM, the V_max_ values were 0.033, 0.138, 0.207, 0.303, and 0.493 nmol/min/mg, and the intrinsic clearance values (CL_int_ and V_max_/K_m_) were 1.3, 9.4, 13.5, 14.6, and 14.0 μL/min/mg, respectively (Table 2). The data were also visualized in the form of a 3D scatter graph (Fig. 4B).

**Table 2:** Kinetic parameters for xanthine oxidation in HLS9 system. Constants are expressed as the mean ± SE

PLB μM	K_**m**_ μM	V_**max**_ nmol/min/mg	Goodness of fit R^**2**^	CL_**int**_ μL/min/mg
0	25.2 ± 2.5	0.033 ± 0.001	0.981	1.3
10	14.7 ± 1.6	0.138 ± 0.003	0.983	9.4
20	15.3 ± 1.1	0.207 ± 0.003	0.993	13.5
40	20.7 ± 3.2	0.303 ± 0.010	0.961	14.6
80	35.2 ± 4.6	0.493 ± 0.016	0.972	14.0

### Kinetic analyses for the stimulation of uric acid production of XO monoenzyme system

**Fig. 5:**
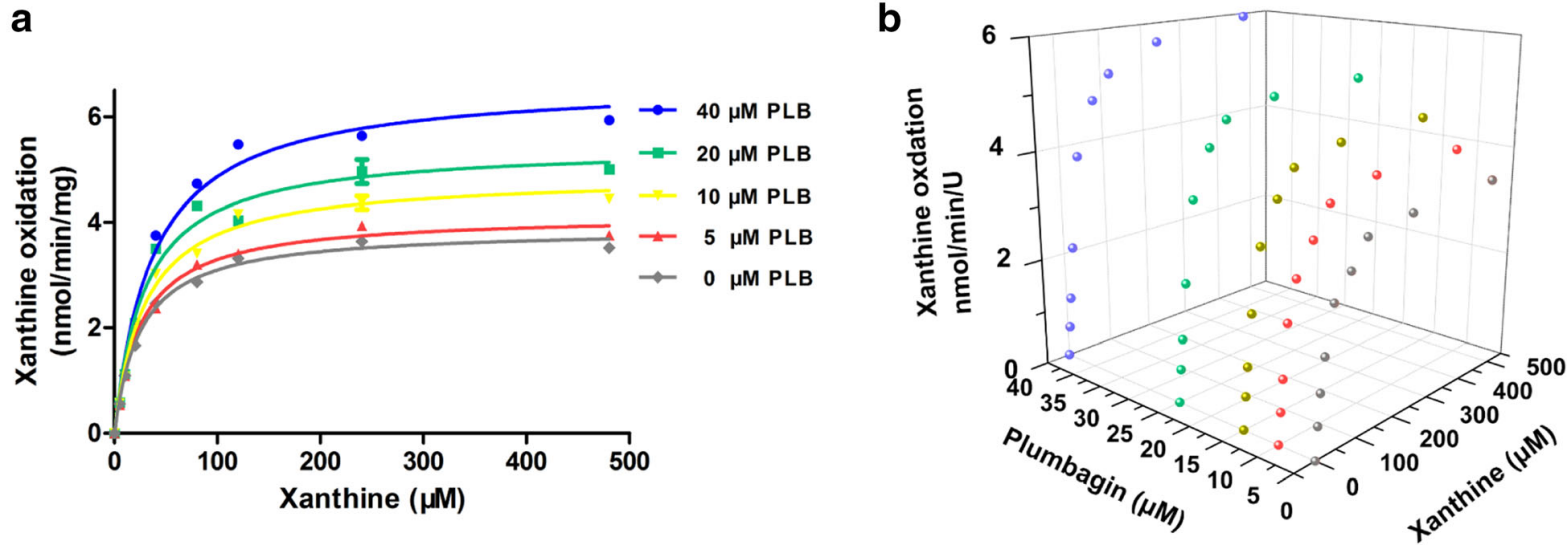
Kinetic analyses for xanthine oxidation stimulation in XO monoenzyme system in the presence of various concentrations of PLB. **A** Kinetics of xanthine oxidation in XO in the presence of various concentrations of PLB. Each point and error bar represents the average and S.D. of duplicate measurements, respectively, and the curves were drawn by fitting the Michaelis–Menten equation to the data. **B** 3D scatter graph for kinetics of activation of PLB on xanthine oxidation in XO monoenzyme system. Each point represents the average of duplicate measurements

To further verify the stimulation of xanthine oxidation, kinetic analyses in XO monoenzyme system were performed as well (Fig. 5A and Table 3). In general, both the K_m_ and V_max_ values increased in a concentration-dependent manner. In the presence of PLB concentrations of 0, 5, 10, 20, and 40 μM, the K_m_ values were 25.3, 25.9, 28.6, 30.2, and 37.0 μM, the V_max_ values were 3.881, 4.147, 4.908, 5.484, and 6.666 nmol/min/mg, and the intrinsic clearance values (CL_int_, V_max_/K_m_) were 153, 160, 171, 181, and 180 μL/min/mg, respectively (Table 3). The data were also visualized in the form of a 3D scatter graph (Fig. 5B).

**Table 3:** Kinetic parameters for xanthine oxidation in XO monoenzyme system. Constants are expressed as the mean ± SE

PLB μM	K_**m**_ μM	V_**max**_ nmol/min/U	Goodness of fit R^**2**^	CL_**int**_ μL/min/U
0	25.3 ± 1.5	3.881 ± 0.058	0.994	153
5	25.9 ± 1.9	4.147 ± 0.080	0.991	160
10	28.6 ± 2.6	4.908 ± 0.1212	0.992	171
20	30.2 ± 3.3	5.484 ± 0.161	0.981	181
40	37.0 ± 3.5	6.666 ± 0.180	0.987	180

### Molecular docking studies

**Fig. 6:**
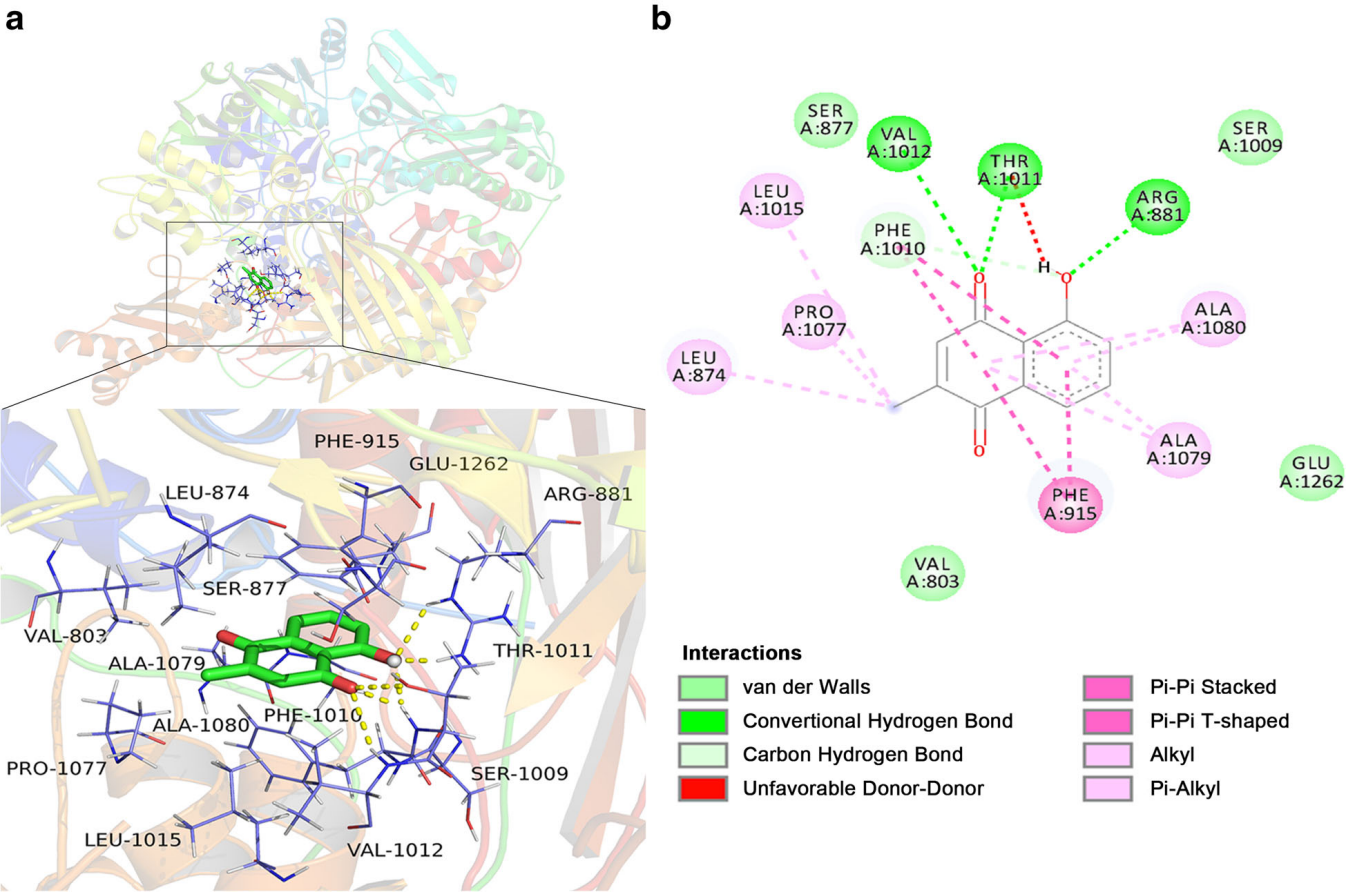
Molecular docking results of the interactions between XO (PDB: 2E1Q) and PLB by AutoDock Vina. **A** Three-dimensional interactions of PLB with the residues of the active site of XO. **B** Docking model of XO with PLB (2D). The lavender linkage indicates a hydrophobic relationship between the amino acid residue and the corresponding ligand, and the light green amino acid residue with no attachments indicates van der Waals interaction between the residue and ligand

In silico studies were performed by using AutoDock to understand the binding modes of the complexes formed between XO (PDB: 2E1Q) and PLB. The docking result showed that the binding free energy is − 7.33 kcal/mol, which is a relatively low free energy, indicating a strong binding strength. Then, we visualized the docking conformation by pyMOL and marked the amino acid residues near PLB, as shown in Fig. 6A. A 2D model of PLB docked with XO is shown in Fig. 6B. The binding conformation shows that PLB can form 6 hydrogen bonds with the residues Arg881, Thr1011 and Val1012, and PLB also has hydrophobic interactions with Ala1080, Ala1079, Leu1015, Pro1077 and Leu874, as well as pi-pi interactions with Phe-915 in the active site.

### Direct binding measurement of PLB to XO by biolayer interferometry analysis

**Fig. 7:**
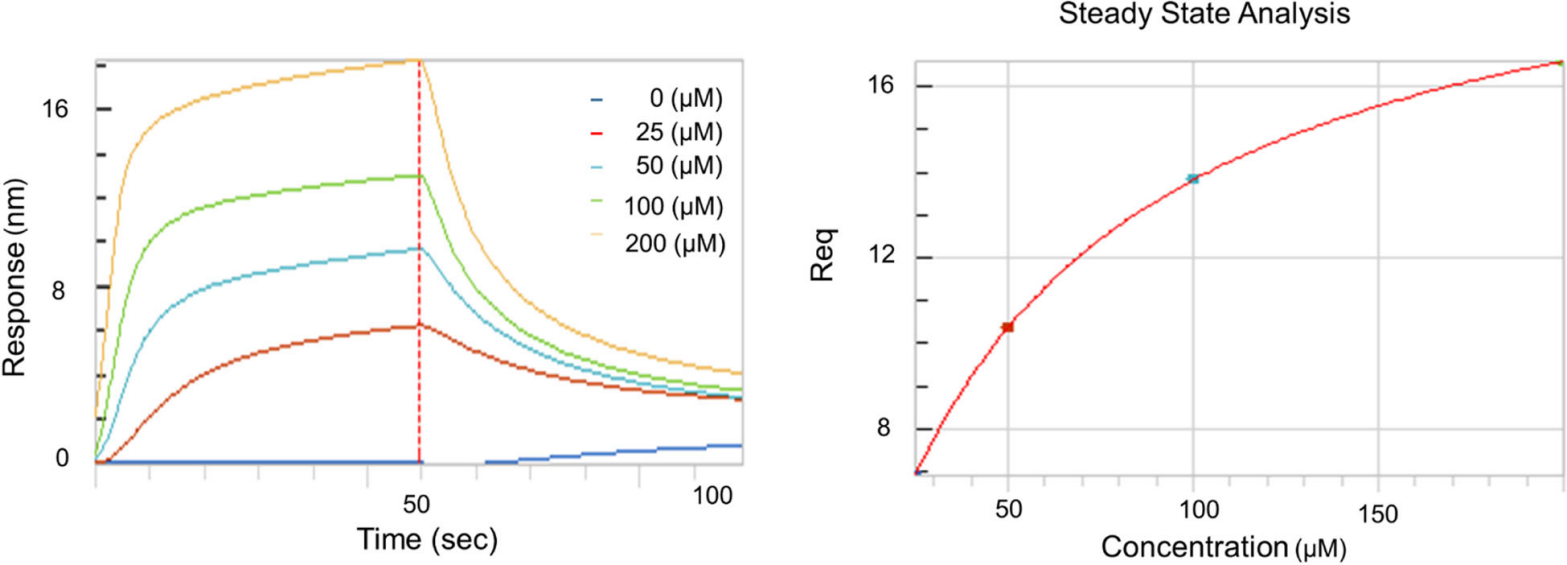
Kinetic measurement of the binding affinity of PLB to XO by BLI assay**.** Kinetic binding sensorgrams of increasing concentrations of PLB from 25 to 200 μM were shown with real-time data acquisition for each step of the kinetic assay. Response/binding (nm) represents the optical thickness of the sensor layer reflected by the spectral shift (1λ) upon the interaction of PLB with XO. The response at steady state where the rate of association is equal to the dissociation and the equilibrium binding signal (Req) indicated by the flattened curve were reached

To determine the binding affinity of PLB to XO, the label-free biolayer interferometry assay, which measures biomolecular interactions was used. First, biotinylated XO was fixed to the tip surface of an SA biosensor, and the interaction between PLB and XO was directly related to the increase in the concentration of PLB (0, 25, 50, 100 and 200 μM). When the binding system reached equilibrium, PLB and XO were dissociated by transferring PLB into a PBS solution containing 5% DMSO. The interaction between PLB and XO was monitored by a ForteBIO Octet Red 96e instrument in real time, and the exact values of the specific binding and dissociation efficiencies of PLB and XO were obtained. The association and dissociative binding curve of PLB and XO is shown in Fig. 7. With increasing PLB concentration, the binding reaction was enhanced, indicating that PLB has a direct binding effect with XO. In addition, the kinetic constant calculated by ForteìBIO biological data analysis software (Table 4) showed that the KD value of PLB was 0.499 μM, the Kon value was 6.72 × 10^+ 02^, and the Kdis value was 3.35 × 10^− 02^, indicating that PLB undergoes a direct and reversible interaction with XO.

**Table 4:** The binding affinity (KD), association rate constant (K_on_) and dissociation rate constant (K_dis_) of PLB to XO

Characters	KD (μM)	Kon (1/Ms)	Kdis (1/s)
PLB	0.499	6.72 × 10^+ 02^	3.35 × 10^− 02^

### Effect of PLB on KM mice

**Fig. 8:**
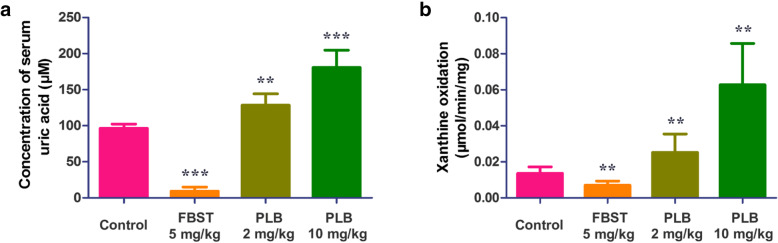
Effect of PLB on KM mice**. A** The concentration of serum UA in KM mice treated with PLB (2 and 10 mg/kg). FBST (5 mg/kg) treatments were set as the negative control group. **B** The activities of serum XO in KM mice treated with PLB (2 and 10 mg/kg). FBST (5 mg/kg) was used as the negative control group. The results are expressed as the mean ± SD of six mice, ***p < 0.001 and **p < 0.01 compared to the control group using a t-test

The activation effect of PLB on XO was further examined using an animal model. KM mice were intraperitoneally injected with PLB (2 and 10 mg per kg b.w.), and an increase in the serum uric acid level and the activity of serum XO compared with that of the vehicle control was observed (Fig. 8).

## Discussion

The unknown toxicity of TCM has always been an important factor restricting its clinical application [16]. Although PLB presents broad pharmacological activity, the obvious toxicities of PLB are not negligible. The PLB-caused imbalance in the antioxidant system is considered to be one of the main causes of hepatic injury [14] but the details remain unclear. Moreover, XO actively participates in the redox process in vivo, and its abnormal increase in activity will lead to oxidative stress injury and metabolic syndrome. Therefore, the interaction between PLB and XO may explain the toxicity of PLB on the imbalance of the antioxidant system.

In this study, when PLB and xanthine were co-incubated with MLS9, HLS9 and XO monoenzyme, PLB displayed accelerating effect on xanthine oxidation, and the effective PLB concentration was indicated to be less than 5 μM. Notably, the intrinsic clearance was 11.2-fold of the control in the presence of 40 μM PLB in HLS9 (Table 2), which is dangerous for the human body. The normal reference interval of uric acid in human blood is 1.5 to 6.0 mg/dL in women and 2.5 to 7.0 mg/dL in men. A uric acid concentration beyond the maximum of the range indicates hyperuricaemia or induces the occurrence of other pathological processes.

To investigate how PLB interacts with XO, molecular docking was then conducted to predict the interaction and binding model between PLB and XO. The results showed strong binding affinity between PLB and XO, with hydrogen bonds, hydrophobic bonds and pi-pi interactions. In addition, biolayer interferometry analysis was performed to study the binding feature, also showing good affinity.

We further verified the effect by PLB-treated mice. Moreover, febuxostat-treated mice were selected as a negative control, although existing compounds, such as potassium oxycyanate [17] and ethambutol [18], have been adopted to induce hyperuricaemia. However, their mechanisms of elevating uric acid levels in vivo are inhibition of uric acid oxidase activity or uric acid excretion, which are not related to XO activity. In contrast, although febuxostat showed a role in decreasing uric acid, but it directly acts on XO [19], which is consistent with the mechanism of PLB increasing uric acid. Therefore, the febuxostat treatment group was eventually selected as the negative control in this study. On the other hand, PLB presents subacute toxicity at doses of 25 mg/kg body weight [20], so the doses of 2 and 10 mg/kg were set as the low-dose and high-dose groups to avoid toxicity in mice. The results showed that after intraperitoneal administration for 3 days, the serum uric acid level of the PLB-treated groups was higher than that of the control group and showed dose dependence, indicating that PLB can increase the serum uric acid level in vivo. To further confirm that the activity of XO is also affected by PLB, we also detected the activity of serum XO. The results showed that compared with that of the control group, the serum XO activity of the PLB treatment groups also increased in a concentration-dependent manner, which is consistent with the results of the in vitro study.

Above all, the present study has demonstrated that PLB can enhance the activity of XO in vitro and in vivo, which may be associated with the apparent toxicity of PLB. On the other hands, it may suggest that we can use XO inhibitors to antagonize the activation effect of PLB on XO activity, thereby reducing the toxicity of PLB to the organism and expanding the therapeutic window. Although the above viewpoint needs to be verified in a further study.

## Conclusions

In summary, our in vitro findings through co-incubation of PLB and xanthine with MLS9, HLS9 or XO outlines a potential source of toxicity for PLB. PLB can significantly accelerate the formation of uric acid by enhancing the activity of XO. Subsequent studies in animal models also showed similar acceleration effects. This may reveal a new potential toxicity of PLB in human beings.

## Data Availability

All data analyzed in this study is available from the corresponding author on reasonable request.

## Abbreviations

XO: Xanthine oxidase
PLB: Plumbagin
HLS9: Human liver S9
MLS9: Mouse liver S9
MSU: Monosodium urate
PBS: Phosphate buffered solution
DMSO: Dimethyl sulfoxide
KM mice: Kunming mice
